# Reverse Gyrase Functions in Genome Integrity Maintenance by Protecting DNA Breaks In Vivo

**DOI:** 10.3390/ijms18071340

**Published:** 2017-06-22

**Authors:** Wenyuan Han, Xu Feng, Qunxin She

**Affiliations:** Archaeal Centre, Department of Biology, University of Copenhagen, Ole Maaløes Vej 5, Copenhagen Biocenter, DK-2200 Copenhagen N, Denmark; xu.feng@bio.ku.dk

**Keywords:** reverse gyrase, *Sulfolobus*, MMS, genomic DNA breakage, genomic DNA degradation, CRISPRi approach

## Abstract

Reverse gyrase introduces positive supercoils to circular DNA and is implicated in genome stability maintenance in thermophiles. The extremely thermophilic crenarchaeon *Sulfolobus* encodes two reverse gyrase proteins, TopR1 (topoisomerase reverse gyrase 1) and TopR2, whose functions in thermophilic life remain to be demonstrated. Here, we investigated the roles of TopR1 in genome stability maintenance in *S. islandicus* in response to the treatment of methyl methanesulfonate (MMS), a DNA alkylation agent. Lethal MMS treatment induced two successive events: massive chromosomal DNA backbone breakage and subsequent DNA degradation. The former occurred immediately after drug treatment, leading to chromosomal DNA degradation that concurred with TopR1 degradation, followed by chromatin protein degradation and DNA-less cell formation. To gain a further insight into TopR1 function, the expression of the enzyme was reduced in *S. islandicus* cells using a CRISPR-mediated mRNA interference approach (CRISPRi) in which *topR1* mRNAs were targeted for degradation by endogenous III-B CRISPR-Cas systems. We found that the TopR1 level was reduced in the *S. islandicus* CRISPRi cells and that the cells underwent accelerated genomic DNA degradation during MMS treatment, accompanied by a higher rate of cell death. Taken together, these results indicate that TopR1 probably facilitates genome integrity maintenance by protecting DNA breaks from thermo-degradation in vivo.

## 1. Introduction

DNA topoisomerases impose changes to the topological states of circular DNA and play important roles in DNA replication, recombination, and repair as well as in RNA transcription [1,2]. Thermophiles encode a unique type of topoisomerase named reverse gyrase (TopR) that introduces positive supercoils to circular DNAs [3]. Because reverse gyrase is the only protein that is exclusively conserved in thermophilic organisms, the enzyme has been implicated in genome stability maintenance in thermophiles [4]. Indeed, genetic studies in *Thermococcus kodakaraensis*, a hyperthermophilic euryarchaeon, show that growth of cells lacking TopR enzyme is significantly retarded at high temperatures (from 65 to 90 °C) [5]. Further, crenarchaea encode two TopR proteins, but attempts to isolate TopR deletion mutants from *S. islandicus* Rey15A was not successful [6], suggesting an essential function for these enzymes in this crenarchaeon. Therefore, TopR enzymes must have important roles in thermophilic life.

Extremely thermophilic organisms have to deal with accelerated levels of spontaneous decomposition of DNA at high temperatures, including deamination of cytosine and base hydrolysis (usually depurination) [7,8,9]. These naturally occurring DNA lesions often lead to the formation of apurinic/apyrimidinic (AP) sites, which eventually yields single strand DNA breaks (SSBs) [7,8,9]. Likewise, base alkylation agents, such as methyl methanesulfonate (MMS), generate DNA lesions that can also be converted to AP sites and SSBs [10]. If not repaired in a timely fashion, AP sites and SSBs induce collapse of DNA replication, giving rise to double strand DNA breaks (DSBs). DSBs are a more severe type of DNA lesion that must be repaired by homologous recombination repair (HRR), an energy-consuming pathway [11,12]. As a result, severe DSB damage on chromosomal DNA often leads to cell death. Therefore, one of the great challenges in thermophilic life is to deal with numerous DNA lesions that result from spontaneous DNA decomposition in order to prevent the formation of DSBs.

Since DNA lesions produced by MMS treatment must be repaired in a similar fashion as DNA lesions generated from spontaneous base decomposition in DNA, MMS treatment provides a useful means for investigating DNA damage repair mechanisms in thermophilic organisms. Indeed, investigation on the effect of MMS on genome integrity maintenance in *Sulfolobus solfataricus* has revealed that TopR1 is subjected to degradation upon a lethal MMS treatment and that MMS-induced TopR1 degradation coincides with genomic DNA degradation [13]. Moreover, it has been shown that the *Archaeoglobus fulgidus* TopR binds to DNA nicks in vitro and inhibits heat-induced degradation of nicked DNA, and the DNA protection mechanism is independent of supercoiling [14]. This raises an interesting question as to whether TopR proteins could protect damaged DNA from thermo-degradation in vivo.

In the article, we employed *S. islandicus*, a genetic model for which very versatile genetic tools have been developed [15], to investigate the function of TopR1 in genome integrity maintenance. We found that MMS treatment induced a series of events including: (a) massive breaks on the sugar-phosphate backbone of genomic DNA, (b) gradual DNA content reduction and TopR1 degradation, and (c) DNA-less cell formation and chromatin protein degradation. Moreover, we constructed a TopR1 knockdown strain (pKD-topR1/E233S1) with a CRISPR-based RNA interference tool (CRISPRi) [16]. The strain exhibits a higher sensitivity to MMS and an early onset of DNA degradation during MMS treatment, indicating that TopR1 protects DNA breaks from thermo-degradation in vivo.

## 2. Results

### 2.1. Determination of Moderate and Lethal Dosages of Methyl Methanesulfonate (MMS) for S. islandicus

**Figure 1:**
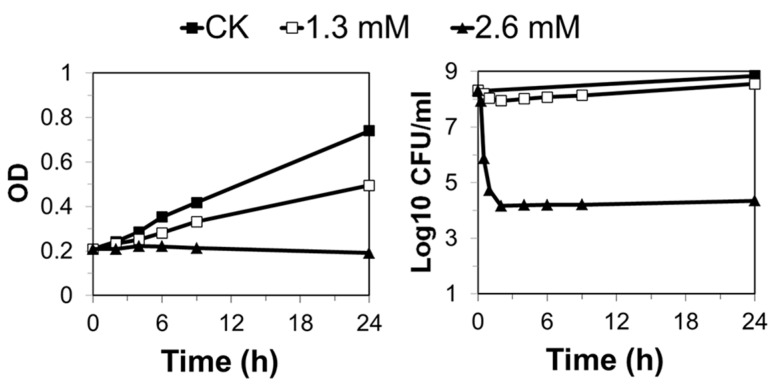
Exponential phase cultures were treated with 1.3 mM and 2.6 mM methanesulfonate (MMS), respectively. The cultures without any treatment were also grown at the same time as control (CK). The optical density (left panel) and cell viability (right panel) were analyzed during the treatment at indicated time points. The data are representative of three independent replicates. (CK, black squares; 1.3 mM, white squares; 2.6 mM, black triangles). OD, the optical density measured at a wavelength of 600 nm; CFU, colony formation unit.

To investigate the effects of MMS on *S. islandicus*, exponentially growing *S. islandicus* cultures were treated with 0.6, 1.3, or 2.6 mM of MMS, and the treated samples were analyzed for culture growth and cell viability. While 0.6 mM MMS did not exert any apparent effect on culture growth (data not shown), 1.3 mM and 2.6 mM MMS yielded growth retardation to the *S. islandicus*. While a strong growth inhibition was observed for the 2.6 mM MMS-treated culture, since the absorbance at 600 nm (A_600_) of the culture stopped increasing immediately after drug addition, only a moderate growth inhibition was attained by the 1.3 mM MMS-treated culture (Figure 1). Furthermore, in the 2.6 mM MMS-treated culture, colony formation units (CFU) declined by more than 500-fold in the first 30 min of drug treatment, and cell viability did not increase in the following 24 h of incubation. In contrast, about a 50% drop in cell viability was observed for the 1.3 mM MMS-treated culture in the first 30 min, and the cell viability started to increase 2 h after drug treatment (Figure 1). Therefore, the two drug concentrations (1.3 and 2.6 mM) represent a moderate and a lethal dosage of MMS for exponentially growing *S. islandicus* cells, respectively.

### 2.2. MMS Treatment Yields Immediate Genomic DNA Breakage and Subsequent DNA Degradation in S. islandicus

**Figure 2:**
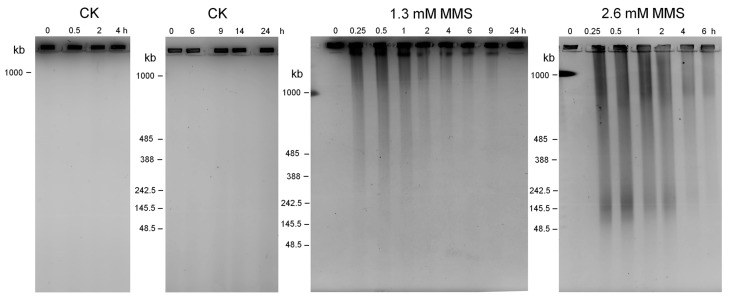
Pulsed field gel electrophoresis (PFGE) analysis of total DNA from untreated culture (CK) and the cultures treated with 1.3 mM and 2.6 mM MMS. Total DNA from about 4 × 10^8^ cells was analyzed for each sample. DNA fragments are visible below the wells in treated samples.

To reveal whether and how MMS induces genome instability in *Sulfolobus*, total DNAs were prepared from cell samples collected during the MMS treatments and analyzed by pulsed field gel electrophoresis (PFGE). As shown in Figure 2, whereas the circular chromosomal DNA of untreated cells was retained in the loading wells over the 24 h incubation, 1.3 and 2.6 mM of MMS induced the accumulation of considerable amounts of genomic DNA fragmentation, which peaked at 15 min after drug treatment, indicating that MMS immediately induced extensive DNA backbone breaks on the chromosome (Figure 2). Noticeably, the higher dosage of MMS yielded more and shorter DNA fragments than the moderate dose, indicating that the number of DNA backbone breaks is correlated to MMS dosage, and, furthermore, a DNA band close to the loading well which could correspond to the linear chromosomal DNA is present in 1.3 mM MMS-treated samples but absent from 2.6 mM MMS-treated samples. Subsequently, the intensity of DNA fragments gradually declined from 2 h in both 1.3 and 2.6 mM MMS treatment. Noticeably, from 6 to 24 h during 1.3 mM MMS treatment, most genomic DNA was retained in the loading wells, similar to that of the untreated samples, suggesting reestablishment of genome integrity after the repair of DNA breaks during the moderate treatment.

**Figure 3:**
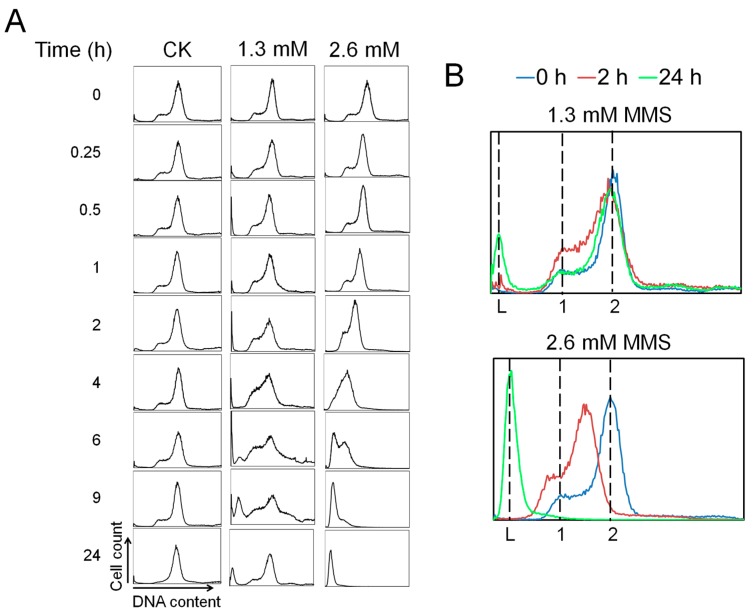
(**A**) Exponential phase cultures were treated with 0 (CK), 1.3 mM or 2.6 mM MMS, and the DNA content distributions were analyzed by flow cytometry at indicated time points; (**B**) Selected results from indicated time points (0 h, blue; 2 h, red; 24 h, green) are directly compared to better show typical changes of DNA content distributions during MMS treatment. Dashed lines marked with “L”, “1”, and “2” indicate DNA-less cells and cells containing one or two copies of genomic DNA respectively. X axis: DNA content; Y axis: cell count. Both X axis and Y axis are shown in linear scale.

Next, we set out to analyze the DNA content distributions in the cells of MMS-treated cultures by flow cytometry (Figure 3). The results showed that DNA content distributions of untreated samples remained unchanged in the first 9 h of incubation. Then, the population of G1 and S phase cells decreased at 24 h, which could reflect that the cells were in a late exponential stage at the time. For the moderate MMS treatment, G1 and S phase cells were accumulated in the first 6 h of incubation, and a small fraction of DNA-less cells appeared at 9 h. By 24 h after MMS addition, most cells showed a DNA content of one or more than one chromosomes. Together, these data suggest that, while the majority of cells were recovered from MMS-mediated DNA damage, some cells underwent cell death. In the lethal MMS treatment, however, the DNA content distribution remained the same in the first 1 h, suggesting that although MMS induce immediate DNA backbone breakage, the DNA breaks were protected from degradation at this time. Then, DNA content gradually decreased as the DNA content peaks moved leftwards from 1 to 4 h during the drug treatment, and eventually a peak corresponding to DNA-less cells occurred at 6 h. Additionally, at 24 h, most cells became DNA-less cells. Thus, we concluded that lethal MMS-induced DNA breaks eventually lead to chromosomal DNA degradation, while DNA breaks after a moderate MMS treatment are largely repairable such that most cells are eventually recovered from the MMS-mediated DNA damage.

### 2.3. MMS-Induced TopR1 Degradation is Prior to Chromatin Protein Degradation

**Figure 4:**
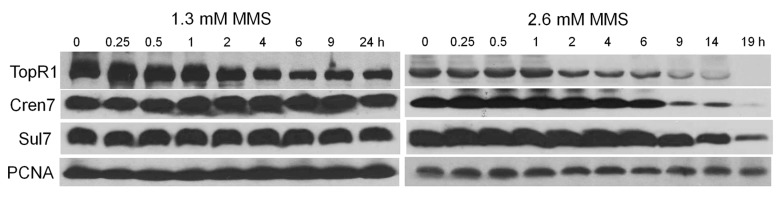
Western blot analysis of the levels of TopR1, Cren7, Sul7, and PCNA3 in the cells treated with 1.3 and 2.6 mM MMS. The sampling time points during the treatments are indicated. Total protein of about 1.3 × 10^8^ cells, given that a culture of OD_600_ = 1.0 contains 1 × 10^9^ cells per mL, was loaded onto the gel.

To investigate the dynamics of chromatin architecture proteins during MMS-induced genomic DNA degradation in *S. islandicus*, we analyzed the level of TopR1, Sul7, and Cren7 in *S. islandicus* cells by western blot during 1.3 and 2.6 mM MMS treatment (Figure 4). In the moderate MMS treatment, Cren7 and Sul7 contents remained more or less the same in the cells whereas TopR1 level decreased from 2 to 6 h. Then, at 9 h after drug addition, TopR1 level started to increase, indicative of full recovery of the cells from the MMS-mediated DNA damage. In the lethal MMS treatment, after the onset of TopR1 degradation at 2 h after drug addition, the level of the protein continuously decreased along with the incubation, which coincided with cellular DNA content reduction. Finally, cellular TopR1 was no longer detectable after 19 h of drug treatment. In addition, lethal MMS treatment also induced chromatin protein degradation at 6 h. The sequential degradation of TopR1 and chromatin proteins suggests that they play different roles in genome stability maintenance in this archaeon.

### 2.4. Knockdown of TopR1 Transcripts Accelerates MMS-Induced Genomic DNA Degradation

**Figure 5:**
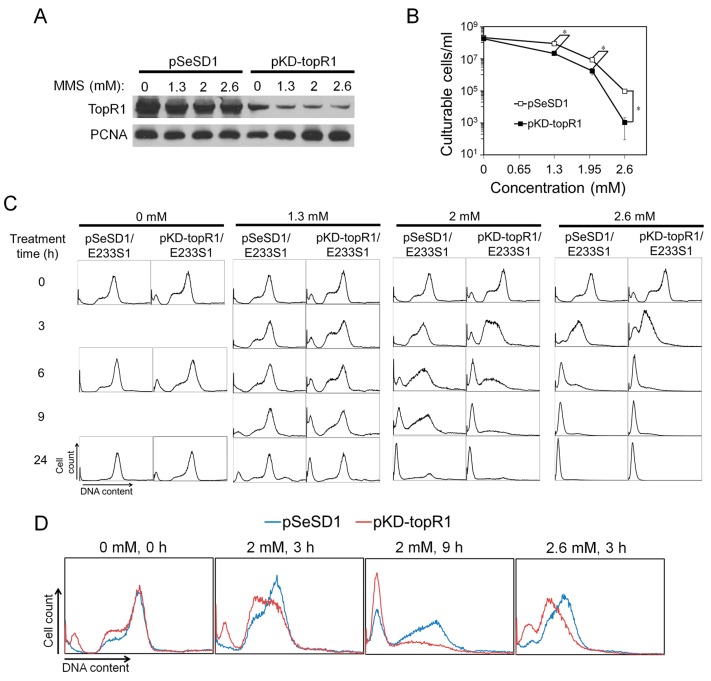
Knockdown of TopR1 accelerated MMS-induced genomic DNA degradation. Reference strain (pSeSD1/E233S1) and the TopR1 knockdown strain (pKD-topR1/E233S1) were treated with 0, 1.3, 2 or 2.6 mM MMS. After 3 h, cellular samples were taken for Western blot analysis of TopR1 and PCNA3 levels (**A**) and cell viability (**B**); (**C**) flow cytometry analysis of DNA content distribution of pSeSD1/E233S1 and pKD-topR1/E233S1 cultures during MMS treatment. Sampling time points are indicated; (**D**) comparison of cellular DNA content of pSeSD1/E233S1 (blue line) and pKD-topR1/E233S1 (red line) during MMS treatment. The MMS concentration and treatment time are indicated. The asterisk represents *p* values less than 0.05. The *p* value was calculated by paired *t* test. The data are representatives of three biological repeats.

To further investigate the functions of TopR1 in genome stability maintenance in *S. islandicus*, we employed a CRISPR-based RNA interference (CRISPRi) method previously developed in our laboratory [16] to reduce the level of TopR1 in the archaeal cells. The CRISPRi plasmid (pKD-topR1) contained an artificial mini-CRISPR locus with a single copy of two spacers that target two different positions on the *topR1* mRNA. After transformation of *S. islandicus* E233S1 with the plasmid, the expression of TopR1 in the transformants (pKD-topR1/E233S1) was analyzed by western blot, and we found that the cellular TopR1 level was indeed much lower than that in the cells containing the reference plasmid pSeSD1 (pSeSD1/E233S1) (Figure 5A). On the other hand, the growth rates of the two strains were similar at around 78 °C (data not shown). These results indicate that reduction of TopR1 did not impair the growth of this archaeon.

These strains were then employed to investigate the roles of TopR1 in genome stability upon MMS treatment. Cultures of pKD-topR1/E233S1 and pSeSD1/E233S1 were treated with different concentrations of MMS for 24 h during which cell samples were taken and analyzed for TopR1 content, cell viability, and DNA content distributions. As shown in Figure 5A, after 3 h of MMS treatment, TopR1 was partly degraded in both pKD-topR1/E233S1 and pSeSD1/E233S1, although the former expressed much less TopR1 protein. In addition, in both strains, increasing MMS concentrations did not lead to further TopR1 degradation at 3 h. Meanwhile, the survival rate of pKD-topR1/E233S1 was about four times lower than pSeSD1/E233S1 during 1.3 and 2 mM MMS treatment and about 90 times lower during 2.6 mM MMS treatment (Figure 5B), indicating that knockdown of topR1 increased MMS sensitivity.

Flow cytometry analysis showed that a small population of DNA-less cells (about 8%) was formed in pKD-topR1/E233S1 cultures even without MMS treatment. Further, its S-phase cell population moderately increased compared to pSeSD1/E233S1 (Figure 5C). The results indicate that, although the growth curve of pKD-topR1/E233S1 was similar to that of pSeSD1/E233S1, reduced TopR1 level also affected genome stability and/or DNA replication in pKD-topR1/E233S1 cells. At 24 h, the population of S phase cells in the pKD-topR1/E233S1 culture also reduced, suggesting that the culture also entered a late exponential stage similar to the pSeSD1/E233S1 culture.

At 24 h after treatment, 1.3 and 2 mM MMS induced a moderate increase of DNA-less cells in the pKD-topR1/E233S1 culture relative to that of pSeSD1/E233S1, while 2.6 mM MMS induced almost 100% DNA-less cells for both strains (Figure 5C). To further reveal the roles of TopR1 in the response to MMS, we compared DNA content distributions between the two strains before DNA-less cell formation, and the results show that the DNA content in pKD-topR1/E233S1 cells was less than that in pSeSD1/E233S1 from 3 h to 9 h during 2 mM MMS treatment and at 3 h during 2.6 mM MMS treatment (Figure 5D), indicating that the cells with a reduced TopR1 level underwent accelerated genomic DNA degradation during MMS treatment.

### 2.5. MMS Resistance of S. islandicus is Concomitant with the Association of TopR1 with Chromosomal DNAs

**Figure 6:**
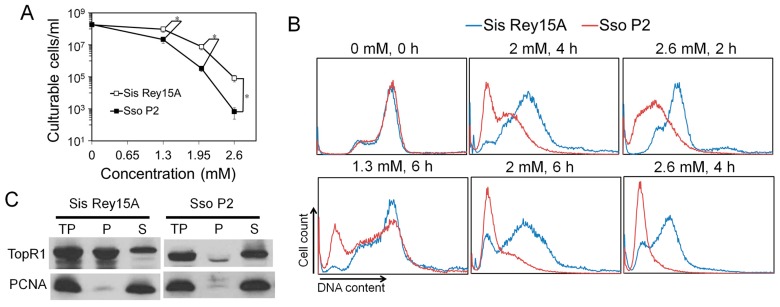
TopR1 was bound to the chromatin part in *S. islandicus*. (**A**) *S. islandicus* Rey15A and *S. solfataricus* P2 cultures were treated with 0, 1.3, 2, or 2.6 mM MMS, and cell viability was analyzed after 2 h of treatment; (**B**) comparison of cellular DNA content of *S. islandicus* Rey15A (blue line) and *S. solfataricus* P2 (red line) during MMS treatment. Selected samples from Supplementary Figure S1 are shown, and the MMS concentration and treatment time are indicated; (**C**) western blots analysis of the subcellular location of TopR1 in *S. islandicus* Rey15A and *S. solfataricus* P2. TP, total protein; P, pellet (chromatin-rich fraction); S, supernatant (cytoplasm fraction). Antiserum against PCNA3 was used as a loading control. The asterisk represents *p* values less than 0.05. The *p* value was calculated by paired *t* test.

Previous research showed that 0.7 mM MMS is a lethal dosage for *S. solfataricus* cells, which is much lower than the lethal dosage (2.6 mM) for *S. islandicus* identified in this work. To verify the results, we compared the MMS sensitivity of *S. solfataricus* and *S. islandicus*. The two species were grown and treated with MMS in the same conditions, and cell viability and DNA content distributions of the treated samples were analyzed. First, during 1.3, 2, and 2.6 mM MMS treatment, *S. solfataricus* exhibited about 4, 23, and over 100-times lower survival rate than that of *S. islandicus*, respectively, confirming that *S. solfataricus* is indeed more sensitive to MMS than *S. islandicus* (Figure 6A). Further, the reduction of DNA content in *S. solfataricus* was also faster than that in *S. islandicus*, indicating that MMS induced more intensive DNA degradation in *S. solfataricus* (Figure 6B and Figure S1). We also noticed that most *S. solfataricus* cells became DNA-less cells after treatment of 2 mM MMS in this study, which is much higher than the lethal dosage for *S. solfataricus* cells reported previously [13]. The difference between the two studies could be due to different growth and treatment conditions in experiments, or unknown strain differences.

It was also reported that SsoTopR1 mainly exists in the cytoplasm of *S. solfataricus* and is not recruited into chromosomes during MMS treatment [13]. Further, AfuTopR1 binds to DNA nicks to protect nicked DNA from thermo-degradation in vitro [14]. Therefore, we analyzed the subcellular location of TopR1 in *S. islandicus* cells as well as in *S. solfataricus* cells. Cell lysates (total protein, TP) of *S. solfataricus* and *S. islandicus* were fractionated into soluble supernatant (S, cytoplasm fraction) and pellet fractions (P, DNA-rich fraction) as previously described [17]. The abundance of TopR1 and PCNA3 in TP, S, and P fractions was analyzed by western blot. As shown in Figure 6C, while most TopR1 in *S. solfataricus* existed in the cytoplasm fraction, more than a half of TopR1 in *S. islandicus* was bound to chromosomes, suggesting that the chromosome-associated TopR1 may be responsible for the higher resistance of *S. islandicus* to MMS.

**Figure 7:**
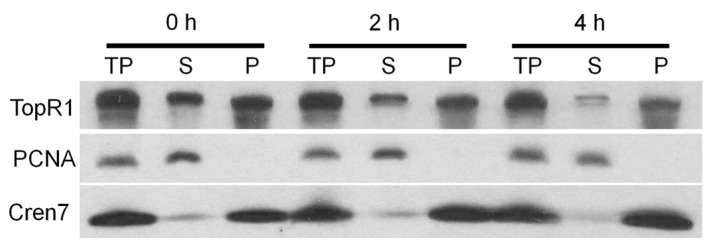
Western blot analysis of TopR1 level from cytoplasm fraction and chromatin-rich fraction during MMS treatment. *S. islandicus* Rey15A cells was treated with 2.6 mM MMS and cells were collected at 0, 2, and 4 h to prepare total protein (TP), cytoplasm fraction (supernatant, S) and chromatin-rich fraction (pellet, P). Antisera against PCNA3 and Cren7 were used as a loading control.

We further analyzed the dynamics of TopR1 protein from the cytoplasm fraction and chromatin-rich fraction during MMS treatment in *S. islandicus* Rey15A. Treated *S. islandicus* Rey15A cells were collected and fractionated as previously described [17], and levels of TopR1 were analyzed in each fraction. The results indicate that TopR1 levels were reduced in both cytoplasm fraction and chromatin-rich fraction (Figure 7), suggesting that MMS-induced degradation occurs to TopR1 from both fractions in *S. islandicus* Rey15A.

## 3. Discussion

In this study, we analyzed the effects of MMS on the genome integrity of *S. islandicus*, a thermophilic crenarchaeon and investigated the functions of TopR1 in genome stability maintenance. Several lines of experimental evidence indicate that SisTopR1 protects MMS-induced DNA breaks from thermo-degradation in a chromosome-associated manner in vivo.

First, we have demonstrated that MMS treatment immediately induces genomic DNA breakage in *Sulfolobus*. MMS does not directly introduce DNA breaks to genomic DNA in yeast and mammalian cells; however, MMS-induced DNA lesions could be converted into DNA breaks by base excision repair (BER) processing or DNA replication collapse [10]. In addition, MMS-induced DNA lesions are heat-labile in vitro and are converted to single stranded DNA breaks (SSBs) at 50 °C; two close SSBs will results in the formation of a DSB [18]. Therefore, the DNA breaks observed in this study can be derived from two main sources: (i) thermo-degradation of MMS-induced lesions at the physiologic growth temperature of *S. islandicus* and (ii) processing of the lesions by BER pathway. Considering the rapid occurrence of genomic DNA breakage, it is more likely that thermo-degradation of MMS-induced lesions constitutes the main source of MMS-induced DNA breaks. As a result, this suggests that MMS can function as a direct DNA break agent in thermophiles.

Second, lethal MMS-induced genomic DNA degradation is evidenced by the decline of DNA fragments as shown by PFGE analysis and gradual reduction of cellular DNA content as shown in flow cytometry analysis. The degradation could be directly triggered by MMS-induced massive SSBs, since an SSB can accelerate thermo-degradation of genomic DNA in vitro: first, supercoiled DNA is not likely to be denatured, while nicks initiate dsDNA denaturation [7]; second, the exposed ssDNA is even more fragile, exhibiting a much higher depurination rate and breakage rate, leading to dsDNA breakage and further degradation [8,9]. More strikingly, we demonstrate that even though MMS induces immediate genomic DNA backbone breakage, cellular DNA content does not start to reduce until TopR1 degradation occurs (Figure 2, Figure 3 and Figure 4), supporting the previous hypothesis that TopR functions in protecting damaged DNA from degradation [13,14]. Further, chromatin protein degradation occurs after TopR1 degradation and coincides with DNA-less cell formation, a common cell death consequence of the treatment of different DNA damage agents (submitted). The sequential degradation of TopR1 and chromatin proteins suggest that after MMS introduces DNA breaks, TopR1 is first subjected to degradation, leading to genomic DNA degradation, which triggers a cell death signal and induces chromatin protein degradation and DNA-less cell formation. The findings also suggest that TopR1 and chromatin proteins play distinct roles in protecting genomic DNA. We reason that TopR1 might bind to the regions that are loosely compacted or not compacted by chromatin proteins. Nevertheless, the detailed mechanisms for MMS-induced DNA degradation and the roles of these chromatin architecture proteins remain to be clarified.

Lastly, the roles of TopR1 in genome stability maintenance have been demonstrated by CRISPRi of TopR1 expression. The strain with decreased TopR1 levels (pKD-topR1/E233S1) is more sensitive to MMS treatment than the reference strain (pSeSD1/E233S1). Further, MMS-induced DNA degradation is strongly accelerated in pKD-topR1/E233S1, suggesting that damaged DNA is prone to thermos-degradation upon an insufficiency of TopR1. The data, together with the fact that MMS induces SSBs at high temperature in vitro [18] and rapid genomic DNA breakage in *Sulfolobus* (Figure 2), indicate that TopR1 functions in the thermo-protection of SSBs in vivo. Conceivably, TopR1 should also protect the DNA breaks derived from spontaneous DNA hydrolysis at high temperatures, which could represent a general function for reverse gyrase in thermophiles.

In addition, knockdown of TopR1 by CRISPRi in *S. islandicus* cells also leads to a moderate accumulation of S phase cells. This either suggests that (1) TopR1 has a role in DNA replication or that (2) the reduced level of protection for spontaneous genomic DNA breaks in TopR1-CRISPRi cells yields DNA replication fork collapse. Indeed, it has been suggested that TopR1 is involved in cell duplication in *S. solfataricus* [19]. On the other hand, we observed that moderate MMS treatment also induces accumulation of S phase cells (Figure 3), supporting that unrepaired DNA breaks arrest DNA replication in TopR1-CRISPRi cells. Currently, it is not possible to distinguish the two possible mechanisms experimentally.

Moreover, the association of SisTopR1 to chromatin and the higher MMS resistance of *S. islandicus* suggest that the DNA protection role of TopR1 is related to its association to DNA. The hypothesis is consistent with the fact that AfuTopR protects nicked DNA possibly by binding to the breaks in vitro [14]. On the other hand, even though TopR1 proteins from *S. islandicus* and *S. solfataricus* exhibit high sequence similarity (Figure S2), most SsoTopR1 protein exists in the cytoplasm fraction. Further, SsoTopR1 is recruited to the chromatin-rich fraction upon ultraviolet (UV) treatment, suggesting translocation of TopR1 upon DNA damage [17]. A similar translocation mechanism might be adopted in *S. islandicus* under normal growth conditions.

TopR1 degradation has been proved to be very specific. It was only observed during MMS treatment, while other stressful conditions, including hydroxyurea (HU) and UV treatment, fail to induce the phenomena [13], even though UV also induces DNA breaks and arrests DNA replication in *S. solfataricus* [20]. We reason that MMS and UV induce different types of DNA breaks: MMS-induced heat-labile DNA lesions are immediately converted to massive SSBs at the physiologic growth temperature of *Sulfolobus*, while UV-induced DNA lesions may be converted to DNA breaks by DNA replication fork collapse. The findings hint that TopR1 is specifically induced by massive SSBs.

Nevertheless, the question regarding the biological meaning of TopR1 degradation remains to be answered. Possibly, TopR1 degradation represents an important step in the archetypal programmed cell death, as hypothesized previously [13], such that TopR1 degradation results in thermo-degradation of damaged DNA, leading to cell death. However, we have shown that TopR1 is degraded to a similar extent from 2 to 6 h in the moderate and the lethal MMS treatment (Figure 4 and Figure 5A). The difference between the treatments is that TopR1 level recovered from 6 to 24 h during moderate MMS treatment but continuously decreased upon lethal MMS treatment. Therefore, we assume that TopR1 degradation also plays a role in DNA repair to increase the local access to DNA repair machineries of the damaged site upon moderate treatment similar to that in Eukaryotes [21,22]. Indeed, TopR-coated dsDNA exhibits limited accessibility in vitro [14]. Furthermore, the two hypotheses are not mutually exclusive: upon moderate MMS treatment, TopR1 degradation increases the local access to DNA repair machineries and facilitates DNA repair and cell survival; upon lethal MMS treatment, DNA repair machineries may fail to repair massive DNA breaks in a timely fashion. As a result, the decrease of cellular TopR1 content would result in exposure of more DNA backbone breaks from thermo-degradation, leading to cell death.

## 4. Materials and Methods

### 4.1. Construction of the TopR1 Knockdown Strain

The strategy for construction of the TopR1 (gene locus: SiRe_1581) knockdown plasmid was similar to that described in [16]. Specifically, we chose two unique sequence regions preceded by a GAAAG motif at the 5′ end from the antisense strand as spacers to abolish DNA cleavage activity [23]. The oligonucleotides for them are shown in the Supplementary Table S1. The spacer fragments were prepared by annealing and extension of each pair of oligonucleotides. Then, the two spacer fragments were mixed and the mixture was used as a template for further amplification with S1-forward and S2-rev-SalI as primers. The obtained fragments were inserted into pSeSD1, an expression vector widely used in *S. islandicus* [24], between StuI and SalI. Then, the generated plasmid was transferred into *Escherichia coli* DH5α, and the colonies were screened by sequencing. At last, a plasmid containing one copy of each spacer was transferred into *S. islandicus* E233S1 and three transformants (pKD-topR1/E233S1) were picked and grown for further analysis. In the meantime, pSeSD1 was also transferred into the genetic host as control (pSeSD1/E233S1).

### 4.2. Culture Growth and Treatment

The genetic host, *S. islandicus* E233S1, was grown in SCVU medium, containing 0.2% sucrose (S), 0.2% Casamino acids (C), 5 µL/mL of vitamin mixture solution (V) and 20 µg/mL uracil (U), as describe previously [25], while other stains, including pSeSD1/E233S1, pKD-TopR1/E233S1, *S. islandicus* Rey15A, and *S. solfataricus* P2 were grown in SCV medium that lacks uracil compared to SCVU. Before treatment, the cultures were grown at exponential phase for at least 72 h, and then early exponential phase cultures (The optical density at a wavelength of 600 nm is about 0.2) were supplemented with MMS up to the indicated concentrations. Aliquots without the drug were set as controls. During treatment, the cultures were grown at 78 °C in the dark and the samples for OD measuring, cell viability assays, flow cytometry, PFGE, and western blot were taken at indicated time points.

### 4.3. Cell Viability

Cells were collected from 1 mL of untreated or treated cultures by centrifugation and resuspended in 1 mL of fresh medium of the same composition. Then, resuspended cells were diluted by 10^2^ and 10^4^ times, and for each dilution as well as the original cell suspension, 10 µL and 100 µL of cell suspension were mixed with pre-warmed top layer gel and spread onto SCV plates as previously described (SCVU for *S. islandicus* E233S1) [25]. The plates were incubated at 75 °C for 6 days; then, plates with suitable colony densities were selected for colony counting. The cell viability was determined in three independent experiments.

### 4.4. Flow Cytometry

To analyze DNA content and cell size distributions, 300 µL of samples was taken from a culture to which 700 µL of absolute ethanol was added. The samples were stored at 4 °C for at least 12 h to fix cells. Then, fixed cells were collected by centrifugation at 2800 rpm for 20 min and washed with 1 mL of 10 mM Tris-NaCl, pH 7.5, and 10 mM MgCl_2_. Cells were collected again and resuspended in 140 µL of staining dye containing 40 µg/mL ethidium bromide (Sigma-Aldrich, St. Louis, MO, USA) and 100 µg/mL mithramycin A (Apollo chemical, Tamworth, UK). Stained cell samples were analyzed by an Apogee A40 cytometer (Apogeeflow, Hertfordshire, UK) equipped with 405 nm laser. A dataset of at least 60,000 cells was collected for each sample.

### 4.5. Western Blot

Cells from 10 mL of the untreated or treated cultures were collected and resuspended in bufferA (50 mM Tris-NaCl, pH 8.0, 100 mM NaCl). Then, 5× SDS-loading buffer was added into the solution, followed by heating at 95 °C for 10 min, to disrupt the cells. The final volume of the samples was calculated according to the optical density to make sure that the number of cells for each line was same. Then, the proteins were separated by sodium dodecyl sulfate polyacrylamide gel electrophoresis (SDS-PAGE) and transferred to a polyvinylidene difluoride (PVDF) membrane (Bio-Rad, Hercules, CA, USA) and the membrane was incubated with antibodies for Cren7 [26], Sul7d [27], TopR1, and PCNA [28]. Afterwards, the membrane was incubated with anti-rabbit horseradish peroxidase (HRP) Secondary antibody (Thermo Fisher Scientific, Waltham, MA, USA) and the enhanced chemiluminescence (ECL) western blot substrate (Thermo Fisher Scientific, Waltham, MA, USA) was used to detect the signals. The antiserum against TopR1 was raised in rabbits using purified recombinant TopR1 protein.

### 4.6. Pulsed-Field Gel Electrophoresis (PFGE)

About 6 × 10^9^ cells (given that 1 mL culture of OD_600_ = 1.0 contains 10^9^ cells) were collected from 30 mL of untreated or treated *S. islandicus* culture by centrifugation. The cells were washed with 50 mL of TEN buffer (50 mM Tris-Cl, pH 8, 50 mM EDTA, pH 8, 100 mM NaCl) and resuspended in 150 µL prewarmed TEN buffer (37 °C). Then, the cell suspension was mixed with 150 µL of 0.8% low-melting-point agarose (precooled to 37 °C) and transferred to a plug mold with 85 µL per well. After solidification at 4 °C for 30 min, the plugs were released from the mold into a 1.5 mL microfuge tube containing 1 mL of NDS solution (0.5 M EDTA, 0.12% Tris, 0.55 M NaOH, pH 8.5, 1% *N*-lauroyl sarcosine sodium salt) and 1 mg/mL proteinase K (Sigma-Aldrich, St. Louis, MO, USA) and incubated for two days at 30 °C. The old proteinase K-containing NDS buffer was replaced by a fresh solution of the same composition after 1 day’s incubation. The plugs then were washed three times with NDS and stored at 4 °C. For PFGE, about one fourth of a plug was transferred into each well of an agarose gel, and electrophoresis was conducted with a CHEF-DR III pulsed-field electrophoresis system (Bio-Rad, Hercules, CA, USA) under the running condition of 3.5 V/cm with the switch time from 5 to 100 s for 40 h. Then, the gel was stained with a nucleic staining dye SYBR gold (Thermo Fisher Scientific, Waltham, MA, USA), and the image was captured by using Typhoon FLA 7000 (GE Healthcare, Chicago, IL, USA).

### 4.7. Isolation of DNA-Rich Fraction

The procedure to isolate DNA-rich fraction from *Sulfolobus* was modified from that described in [29]. Specifically, about 8 × 10^9^ cells from different *Sulfolobus* species were harvested by centrifugation, washed with extraction buffer (50 mM Tris-HCl pH 7, 15 mM MgCl_2_, 50 mM NaCl, 1 mM DTT, 0.4 M sorbitol) and resuspended with 400 µL of the same buffer. Then, half volume of the cell solution was mixed with 5× SDS-loading buffer (total protein, TP), while the rest was supplemented with 0.5% Triton X-100, placed on ice for 30 min, and subjected to centrifugation at 13,000 rpm for 10 min at 4 °C. The supernatant (S, cytoplasm fraction) derived from the centrifugation was then mixed with 5× SDS-loading buffer, while the pellet (P, DNA-rich fraction) was resuspended with 200 µL of extraction buffer and also mixed with 5× SDS-loading buffer. At last, all three fractions were heated at 95 °C for 10 min and 10 µL of each fraction were run on SDS-gels and analyzed by western blot.

## 5. Conclusions

Lethal MMS treatment induces immediate genomic DNA breakage, followed by genomic DNA degradation. The latter co-occurs with TopR1 degradation. Further, cells with a lower TopR1 level are more sensitive to MMS and undergo accelerated genomic DNA degradation during MMS treatment. Together, the data indicate that TopR1 could protect DNA breaks from degradation in vivo.

## Supplementary Materials

Supplementary materials can be found at www.mdpi.com/1422-0067/18/7/1340/s1.
