# Supplementary material for: Reverse Gyrase Functions in Genome Integrity Maintenance by Protecting DNA Breaks In Vivo
Source: Int J Mol Sci. 2017 Jun 22;18(7):1340. doi: 10.3390/ijms18071340 (PMC5535833; doi:10.3390/ijms18071340)
Supplement: Supplementary file 1 [file ijms-18-01340-s001.docx]

**Table S1. Oligonucleotides used in this work**

| **Oligonucleotide** | **Sequence (5’->3’)** |
| --- | --- |
| S1-forward | GCTAATCTACTATAGAATTGAAAGAATATTAATCAGATCAGATAAATTGTTAAACTCTTT |
| S1-rev-SalI | CGCAGTCGACTTTCAATTCTATAGTAGATTAGCGGATAAAGAGTTTAACAATTTATCTG |
| S2-forward | GCTAATCTACTATAGAATTGAAAGACCAGTAGTTAAATTAGCGCCATAAAGTCTAGATGT |
| S2-rev-SalI | CGCAGTCGACTTTCAATTCTATAGTAGATTAGCCAGAACATCTAGACTTTATGGCGCTA |
|  |  |


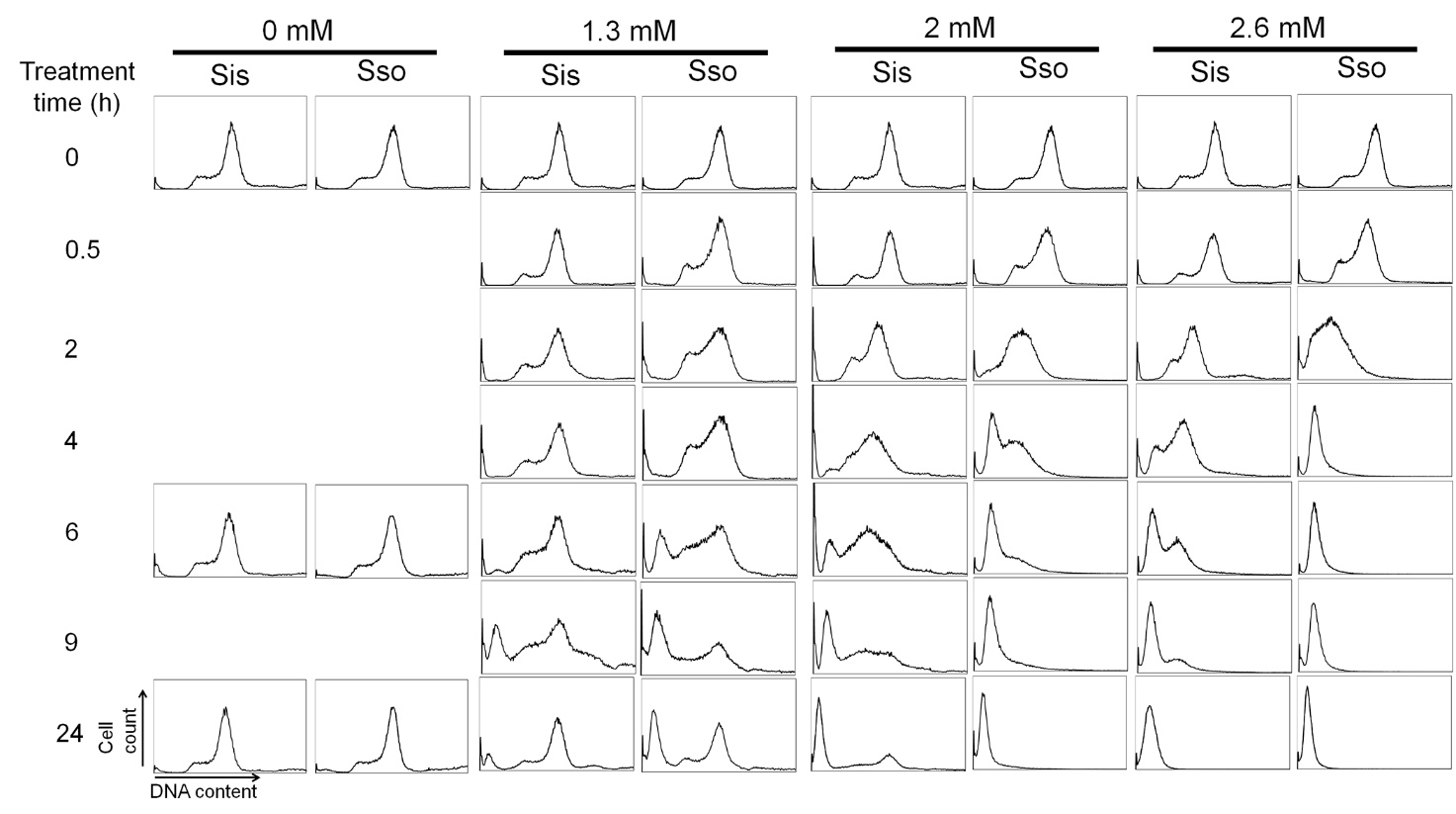


**Figure S1**. The figure is related to Figure 6B. Flow cytometry analysis of DNA content distributions in *S. islandicus* Rey15A and *S. solfataricus* P2 during MMS treatment. The concentrations of MMS and the sampling time points were indicated.


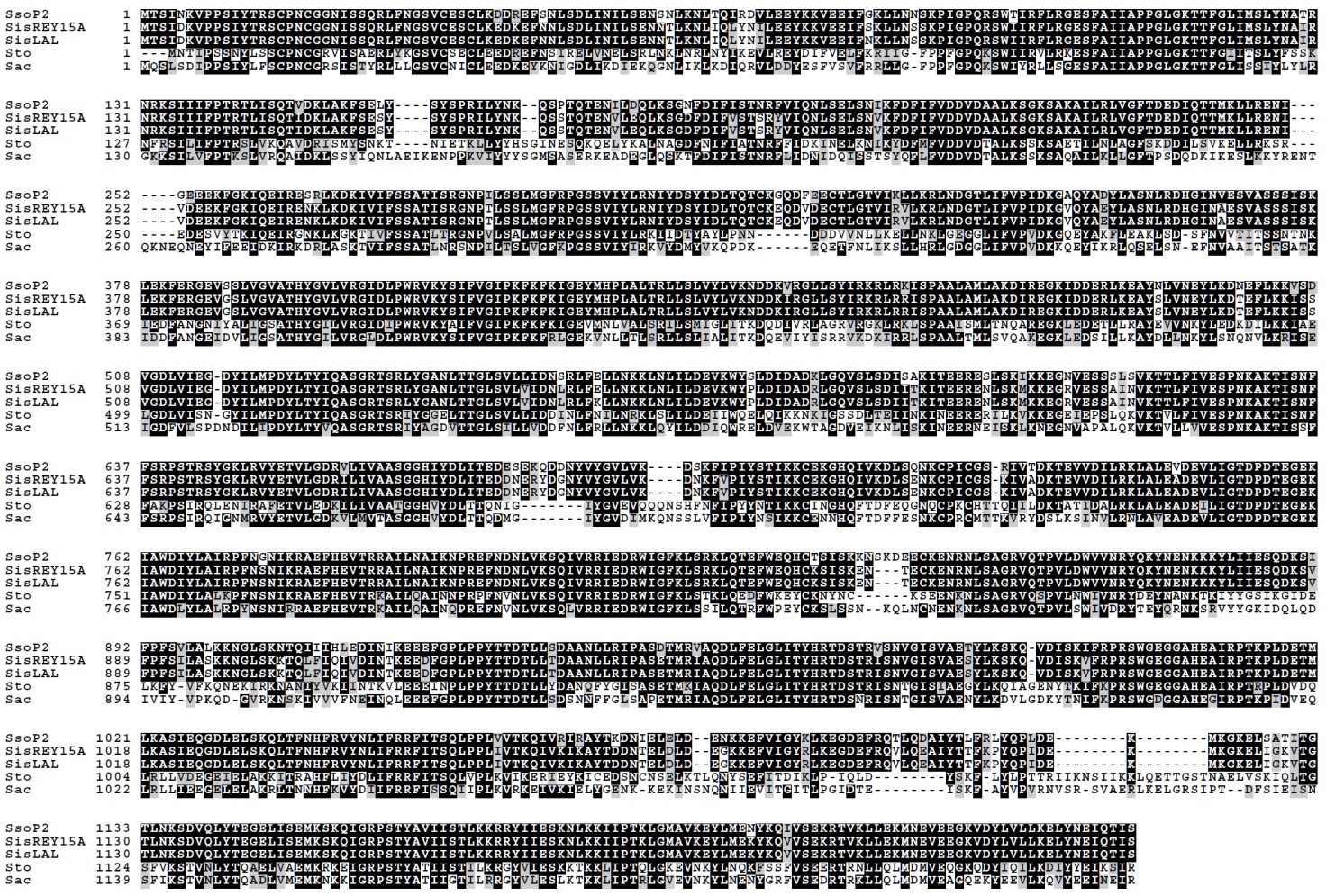


**Figure S2**. Sequence alignment of TopR1 proteins from *S. islandicus* Rey15A, *S. islandicus* LAL14/1, *S. solfataricus* P2, *S. acidocaldarius* DSM 639 and *S. tokodaii* str. 7. The alignment was made by Clustal Omega (<http://www.ebi.ac.uk/Tools/msa/clustalo/>) and the results were illustrated using a boxshade program (http://www.ch.embnet.org/software/BOX_form.html) with a threshold of 0.5.
